# Is There Just One Type of Multisport Pathway? A Scoping Review of Multisport Engagement in Early Athlete Development

**DOI:** 10.1186/s40798-023-00644-x

**Published:** 2023-10-18

**Authors:** Gillian Ramsay, Alexandra Mosher, Joseph Baker

**Affiliations:** 1 Faculty of Kinesiology and Physical Education, University of Toronto, Toronto, ON Canada; 2 School of Kinesiology and Health Science, York University, Toronto, Canada

**Keywords:** Specialization, Diversification, Sampling, Deliberate practice, Deliberate play, Youth development

## Abstract

Multisport engagement is positioned as the antithesis to specialization within youth development pathways. However, different terms are used to describe the multisport pathway, which may create confusion regarding what the pathway should look like. This review investigated all published research examining the multisport pathway, with a focus on terminology, and how different terms have led to varying interpretations of this research. Four databases were searched for all peer reviewed studies published up until December 2021. All included papers were full text, in English, and focusing on multisport athlete engagement. In total, 1974 abstracts were screened for inclusion eligibility, resulting in 82 articles included within this review. General results showed most studies are empirical (71%, *n =* 58) and looked at athlete development pathways using retrospective questionnaires aimed at investigating the specific pathway to sporting excellence. However, despite the consensus that multisport athletes play many sports in their lifetime, there is little investigation into when and the level of intensity (play versus practice) at which these sports are being played. Further, inconsistencies in the terminology used to describe this pathway have made it difficult to understand potential mechanisms that lead to any positive or negative effects. It is recommended that differences between the key terms of *diversification* and *sampling* are clarified and should not be regarded as synonymous as they may represent different paths within multisport development based on varying levels of intensity of play and practice.

## Key Points

- This scoping review considers all published work considering the multisport pathway in athlete development.
- It was found that there are multiple terms being used to describe the multisport pathway with different operationalizations of those terms. This is turn leads to confusion regarding what the multisport pathway looks like and what mechanisms make this pathway potentially more beneficial to youth athletes.
- This research proposes a distinction between the commonly used multisport terms *sampling* and *diversification* by taking into consideration the different intensity levels of multisport engagement athletes may be involved in.

## Introduction

The increasing attention to high-level sporting engagement in youth and adolescence is reflected in the creation of events like the Youth Olympics, which focuses on competitors between 14 and 18 years of age, and the Little League World Series, which involves young baseball players between the ages of 10 and 12 years of age. Although there is a consensus that athletes will eventually need to specialize at some point in their careers, the best time for this to occur is still a topic for discussion [1].

Parallel to this social interest in high level youth and adolescent sport, there has been a substantial increase in research related to youth and adolescent involvement in sports, including the risks of early sport specialization on the health and long-term development of elite athletes.

Several risks related to sport specialization have been identified, including increased prevalence of physical injury and athlete burnout [2–5], poor mental health [6], and decreased adult participation in physical activity in later years due to either a lack of skills in different sports [7], or an aversion to sport based on their specialized past [7, 8]. However, despite consensus statements from both the American Orthopaedic Society for Sport Medicine and the International Olympic Committee recommending against early sport specialization [9, 10] solutions to this issue, in youth sport contexts that are becoming more specialized and professionalized, are not clear.

Many general models of youth sport and long-term athlete development emphasize an early period of engagement in multiple sports based on the presumption that this type of participation has benefits for long-term skill development and participation that are not found in more specialized approaches. In contrast to a single sport pathway during youth, considerable research emphasizes the potential value of a multisport pathway [4, 7, 11, 12]. This approach is regularly proposed as more beneficial for long-term athlete development in terms of increased recreational participation in sport as children age [13]. However, ‘how’ and ‘why’ it may be more beneficial are not clear. For example, there is evidence that playing multiple sports is no better in terms of managing athlete training load [14, 15], and there is mixed evidence for the relationship between play-based activities and intrinsic motivation in competitive athletes [16, 17].

The lack of clarity about the mechanisms of any positive effects may come from poor measurement precision in this area. For instance, recent examinations of the evidence against early specialization have noted several methodological and conceptual shortcomings, many of which are equally relevant for understanding the value of the multisport pathway in early athlete development. A recent systematic review by Mosher et al. [18] considered how early specialization has been measured and conceptualized and noted a lack of consistent understanding and application of the term *early specialization*. It was found that different interpretations of the term used in prior research may have limited overall understanding of the relationships under examination. Moreover, most of the studies (63%) in the review were non-data driven, calling into question how such strong recommendations against early specialization could be achieved. In addition, work has used either correlational or retrospective designs, which are insufficient for understanding causal relationships, generally reflecting a lack of attention to identifying the specific mechanisms associated with any potential negative consequences [1].

While playing multiple sports as a youth is often positioned as the antithesis to “early specialization” [13, 19–22], the evidence base shows similar signs for concern. More specifically, there are inconsistencies in how the multisport pathway has been defined. Voigt and Hohmann [23], for example, defined the multisport pathway as “diversified involvement in a range of other sports with later specialization” (p. 39), and Baker [11] similarly described it as “involvement in a number of different sports before specializing in later stages of development” (p. 85). However, Güllich [20] defined it more generally as “reduced early sport-specific practice/training” (p. 2281), which is similar to Santos et al.’s [24] definition of “practiced more types of sports during their sporting career” (p. 1763), and Travassos et al.’s [25] with “participation in different sports” (p.1). In contrast, Ford and Williams [26] were more specific, defining it as “a large number of hours in a number of sports coupled with a low number of hours in the sport in which they eventually achieve expert performance” (p. 710). However, in more recent studies, it is more common to see definitions being linked to enjoyment and the idea of play versus practice like Thomas and Güllich’s [27] definition of “little sport-specific coach-led practice and extensive self-led play in various sports through childhood and subsequent specialization at 13-15yrs of age” (p. 1121), and Andrew et al.’s [16] with “sample multiple sports through extensive peer-led play in childhood, with little coach-led practice and specialization in a sport occurring later” (p. 1). All this to say, the lack of consensus on how the multisport pathway has been defined within prior research is an important limitation of this field.

Similar inconsistencies exist in how key terms are positioned in research and theory. Common terms include *diversification* or *sampling*, the latter being especially common in work framed using the Development Model of Sport Participation (DMSP; [13]). In the DMSP, the sampling stage occurs between 6 and 12 years of age and reflects the tendency for many future elite athletes to engage in multiple sports in a play-like manner. Advocates argue there are many benefits from the sampling pathway such as the increased likelihood of continuing physical activity into adulthood due to increased enjoyment, fewer overuse injuries, as well as increased development of social and life skills like teamwork, communication, and respect [12]. Sampling is tied to the concept of deliberate play, defined as “activities in which children participate because they are inherently enjoyable, but could nonetheless contribute to the development of expertise” (p. 8). This is opposed to the single sport pathway, which emphasises deliberate practice, defined as “…requires a high amount of concentration, is not inherently enjoyable, and must be carried out over time” (p. 7).

However, despite the tendency for research on *sampling* and *diversification* to be grouped together to define the multisport pathway, it is not clear that these terms are synonymous. As this field continues to evolve and develop, it is critical that measures and frameworks become more precise; otherwise, drawing distinct conclusions and making clear recommendations are not possible. As the review by Mosher et al. [18] noted regarding terminology around early specialization, it is possible the ambiguity between sampling and diversification has limited our understanding of this phenomenon.

Recently, several systematic reviews have explored elements of the evidence related to early specialization [18, 28, 29], but there has been little exploration of research on the multisport pathway. An exception is the recent scoping review by Murata et al. [30], which looked at the evidence base of *sampling* between sports regarding athlete development and any knowledge gaps for future research. The review found that multisport athletes were not hindered in their potential to become high-performance in their sport, but that few studies investigated multisport participation and personal development of athletes, a key pillar of *sampling* as laid out by Côté et al. [13]. Moreover, the review called for more detailed studies to try and not only pinpoint the positive mechanisms at work within *sampling*, but to try and come up with best practices for athletes in this pathway. Finally, Murata et al. [30] used *sampling* as their key term and touched briefly on different terminology within the multisport pathway concluding that there are no patterns as to why different terms exist and researchers can use either *diversification* or *sampling* in future multisport research, as long as they acknowledge different labels exist. While this is clearly important, it would be valuable to determine how these terms have been operationalized in previous studies. Clear terminology and definitions within both pathways are needed to identify the mechanisms that make multisport participation less harmful than specialization for youth athletes.

This review aims to investigate all research examining the multisport pathway to date. More specifically, a scoping review was conducted to identify peer reviewed journal articles published on the topic of multisport engagement in hopes of determining a) which terms have been used the most in prior work to define the multisport pathway and b) how those terms have been individually operationalized.

## Methods

The Preferred Reporting Items for Systematic Reviews and Meta-Analyses extension for scoping reviews (PRISMA-ScR) checklist [31] was used to guide the exploration of literature pertaining to diversification in sport. The search was initially conducted in the summer of 2019 with an additional search completed in December 2021 to identify any additional articles published in the two years prior. The following four databases were searched: (1) Web of Science, (2) SPORTDiscus, (3) Scopus, and (4) The Sport Education and Medicine Index. The first search looked for articles from as early as possible until June 2019, with the second search looking for articles published from January 2019 until December 2021.

### Search Strategy and Study Selection

Given the variability in how multisport has been defined, searches were performed using a range of key words. Boolean phrases were used along with wildcard symbols and key terms to create four searches per database: (1) diversification* AND (*youth* OR *early* OR *children* OR *adolescents*) AND sport*, (2) multi-sport* AND (*youth* OR *early* OR *children* OR *adolescents*), (3) *late* AND (specialization* OR specialisation*) AND sport*, and (4) *early* AND sport* AND sampling*.

### Inclusion and Exclusion Criteria

The first exclusion phase looked at abstract and title only, and the second required reading the full text of the article. The inclusion criteria for both phases were: (a) peer reviewed studies with full English text availability, (b) a research focus on the multisport pathway in a sporting context (any studies focused solely on single-sport athletes were removed as were studies looking at within-sport diversification where athletes were considered multisport based on different events within the same sport), and (c) athlete focused (athletes did not have to be the sample, but the paper had to be focused on them). A total of 82 articles were submitted for final analysis to be used within this review. Two authors (GR and AM) agreed on the search terms and inclusion criteria for the study, no automation tools were used during the exclusion process, and one author (GR) performed the exclusion for both phases.

### Data Extraction

Articles were considered in two categories. The first included position statements, commentaries, and reviews, while the second contained all empirical studies. When assessing each empirical study, a system for categorizing key variables was created. Each article’s background, introduction and scope were examined relative to three primary variables: (a) having an athlete’s development pathway under investigation within the aim of the paper; (b) whether a specific term was used to describe the sport development pathway under examination (e.g., *sampling* or *diversification*) and the subsequent definition of that term, and (c) whether a specific developmental framework was referenced within the paper. In addition, the methodology sections provided information on sample size, type, age, country, sport, and data collection method. This section also provided data regarding how multisport was defined (yearly, seasonally, or lifetime) and information about intensity of practice/training (days/wk and hours/wk). Finally, the results and conclusion sections of each article were analysed for conclusions related to the multisport pathway. GR performed all data extraction with AM and JB reviewing the final table. No automation tools were used in this process.

## Results

**Fig. 1:**
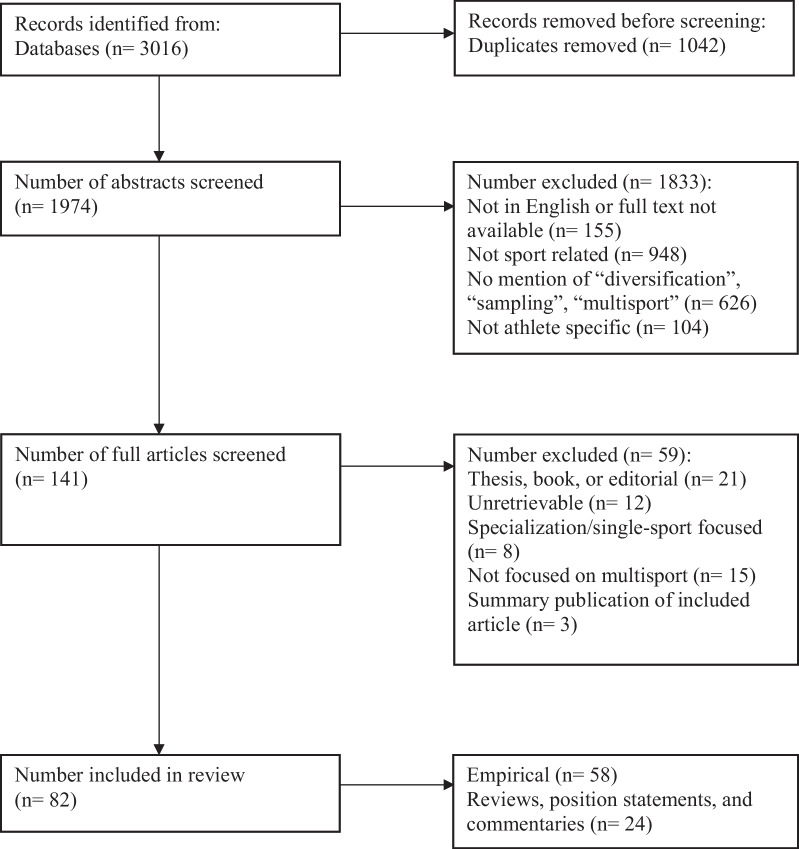
PRISMA flow chart showing inclusion and exclusion of studies

The initial search identified 1,974 papers once duplicates were removed. The first exclusion phase looked at abstract and title only and resulted in 141 studies that were then submitted to a second phase, which required reading of the full text. A final total of 82 articles were submitted for final analysis in this review (Fig. 1). Of the 82 papers comprising this review, 21% (*n =* 17) were position statements or commentaries, 9% (*n =* 7) were reviews, and the remaining 70% (*n =* 58) were empirical research studies. A data set of all 82 studies contained within this review is available on the Open Science Framework (ID number: R6W3E).

### Reviews, Position Statements and Commentaries

Amongst the non-empirical studies in this investigation were seven systematic reviews that included between eight [32] and 115 [33] articles. Most of the earlier reviews had similar objectives (i.e., to look at the talent development pathways of elite athletes), while more recent work (e.g., Murata et al. [30]) focused on questioning specific development pathways. All reviews concluded that playing multiple sports was a viable alternative to specializing in one and/or specializing in one sport was not a requirement to become a high-performance athlete [11, 19, 33, 34]. In addition, Murata et al. [30] showed that most of the studies on multisport athletes focused on the outcome of athletic performance rather than personal development, one of the main postulates of the sampling pathway within the DMSP.

Considering the 17 position statements/commentaries on the multisport pathway, the same conclusion (i.e., that playing multiple sports can lead to expertise) was common. As echoed above there is little attention outside of performance, with no articles reflecting on how the multisport pathway could aid in mental health and personal development in children as implied by the work by Côté and colleagues [12]. In addition, most articles have considered the topic of multisport engagement as a broad topic of discussion focusing on reviewing the positives and negatives of the multisport versus single sport pathway from a country wide perspective [35], or age group perspective [5]. There were three articles [4, 25, 36] that were more narrowly focused and provided discussion on one specific sport (tennis and soccer respectively), while two articles specifically focused on the DMSP [13, 37]. These articles provide valuable insight into the position and opinion of experts regarding the multisport pathway, but as they were not analytically testing new ideas or investigating specific research questions, these non-empirical articles were not analysed any further.

### Empirical Studies

Empirical studies (*n =* 58) made up over two thirds (71%) of the articles identified in the search. The 58 studies (Table 1) were published between 2003 and 2021. Only seven (12%) of the 58 empirical studies used qualitative interviews of either coaches [23, 38, 39], athletes broadly [40–42], or youth athletes in particular [43] to discuss talent development pathways and experiences. Once again, conclusions focused on the theme that coming from a multisport background did not inhibit an athlete’s opportunities of becoming elite.

**Table 1:** All empirical studies (*n =* 58) and their results regarding terminology, operationalisation, and definition regarding the multisport pathway

Study	Term	Definition of Term	Methodology	How was multisport questioned?
Gallant et al. [8]	*Sampling*	"Participating in a variety of sports at a young age"	Questionnaire	Play and practice in main and other sports
Güllich [21]	*Various sports*	"Three stage progression of sampling, specializing and investing"	Questionnaire	Play and practice in main and other sports
Bridge and Toms [44]	*Sampling*	"Individuals engage in a wide range of activities, the majority of which are deliberate play"	Questionnaire	Number sports played
Leite et al. [45]	*Diversification*	"Multiple sport involvement before specializing at later stages of development"	Questionnaire	Number sports played
Coutinho et al. [46]	*Diversification*	–	Questionnaire	Number sports played
Cupples et al. [47]	*Sampling*	"Multiple activity and sport involvement in the early years, with low and high engagement in deliberate practice and play respectively"	Questionnaire	Play and practice in main sport only, and number of other sports
Ford et al. [48]	*Diversification*	"Participate in a large number of hours of deliberate play across a number of sports, but a low number of hours in deliberate practice"	Questionnaire	Play and practice in main sport only, and number of other sports
Ford et al. [49]	*Diversification*	"Engage in a number of different sport activities during childhood"	Questionnaire	Play and practice in main sport only, and number of other sports
Ginsburg et al. [50]	*Sampling*	"Serving the developmental needs of children through enjoyment and participation in various activities"	Questionnaire	Number sports played
Güllich and Emrich [51]	*Diversification*	"Participate in multiple sports during childhood and youth ages"	Questionnaire	Number sports played
Güllich [20]	*Diversification*	"Reduced early sport-specific practice/training"	Questionnaire	Number sports played
Ford and Williams [26]	*Diversification*	"Accumulate a large number of hours in a number of sports coupled with a low number of hours in the sport in which they eventually achieve expert performance"	Questionnaire	Number sports played
Haugaasen et al. [52]	*Diversification*	"Participating in diverse or play activities"	Questionnaire	Number sports played
Güllich [22]	*Sampling*	–	Questionnaire	Play and practice in main and other sports
Hendricks et al. [14]	*Late specialization*	–	Questionnaire	Number sports played
Huxley et al. [40]	*Diversification*	"A greater involvement in a variety of sports before specializing in later stages of development"	Questionnaire	Number sports played
Mendes et al. [53]	*Diversification*	"Intensification of practice/training in one sport later in adolescence"	Questionnaire	Number sports played
Moesch et al. [54]	*Diversification*	"Involvement in different sports as well as a high amount of play-like practice that focuses little on deliberate practice activities"	Questionnaire	Number sports played
Santos et al. [55]	*Diversification*	–	Questionnaire	–
Sieghartsleitner et al. [56]	*Diversification*	"Build a foundation with broad and different experiences from several kinds of sports"	Questionnaire	Play and practice in main sport only, and number of other sports
Thomas and Güllich [27]	*Diversification*	"Little sport-specific coach-led practice and extensive self-led play in various sports through childhood and subsequent specialization at 13–15 yrs of age"	Questionnaire	Play and practice in main and other sports
Nagano and Oyama [57]	*Sampling*	"Participating in more than one (multi) sport"	Questionnaire	Number sports played
Bloom et al., 2021 [58]	*Sampling*	"Young athletes participate in a wide variety of sports at a young age"	Questionnaire	Number sports played
Güllich et al. [59]	*Both sampling and diversification*	–	Questionnaire	Play and practice in main and other sports
Andrew et al. [16]	*Diversification*	"Sample multiple sports through extensive peer-led play in childhood with little coach-led practice and specialization in a sport occurring later"	Questionnaire	Play and practice in main sport only, and number of other sports
Coelho et al. [60]	*Diversification*	"Involvement in several sports during the early years of development in which activities are less structured (i.e. deliberate play)"	Questionnaire	Play and practice in main and other sports
Cowan et al. [61]	*Diversification*	"Continue to participate in other sports to varying degrees alongside the target sport"	Questionnaire	Play and practice in main sport only, and number of other sports
Barth and Güllich [62]	*Diversification*	"Extensive childhood/adolescence multisport deliberate play that is regulated by the participants not the coach"	Questionnaire	Play and practice in main and other sports
Larson et al. [63]	*Sampling*	"Playful participation in multiple sports"	Questionnaire	Number sports played
Ryder et al. [64]	*Diversification*	"Sampling a variety of sports then focusing on a singular sport"	Questionnaire	Number sports played
Stegmann et al. [65]	*Sampling*	–	Questionnaire	Play and practice in main sport only, and number of other sports
Ford et al. [66]	*Sampling*	"Specialisation into a single sport delayed until adolescence"	Questionnaire	Play and practice in main sport only, and number of other sports
Hayman et al. [67]	*Late specialization*	"High quantities of deliberate play activities during the sample years"	Interviews	Number sports played
Countinho et al. [68]	*Diversification*	"Children sample a wide range of sport activities during childhood and experience high levels of deliberate play"	Interviews	Number sports played
Mascarin et al. [69]	*Diversification*	"Practiced other sports without competitive focus"	Interviews	Number sports played
Coutinho et al. [70]	*Diversification*	"Sample a wide range of sporting activities…primarily for enjoyment"	Interviews	Play and practice in main and other sports
Steinl et al. [71]	*Multi-sport*	–	Websites	Number sports played
Macphail et al. [43]	*Sampling*	"Involvement in a range of sports and other activities, experiencing enjoyment and fun, competition, fitness and health benefits, deliberate play and friendships and peer relations."	Observations, interviews, & questionnaires	Number sports played
Storm et al. [42]	*Sampling*	"Sampling a range of sports to then focusing on one”	Interviews	Number sports played
Thomas and Wilson [39]	*Sampling*	"Deliberate play activities and sampling a variety of sports"	Interviews	–
Voigt and Hohmann [23]	*Diversification*	"Diversified involvement in a range of other sports with later specialization"	Interviews	–
Huxley et al. [40]	*Sampling*	"Participation in a variety of sports with the emphasis on deliberate play"	Interviews	Number sports played
Rothwell et al. [41]	*Diversification*	–	Interviews	–
Santos and Tavares [38]	*Diversification*	"Sample a range of sports and develop a foundation of fundamental movement skills"	Interviews	–
Fransen et al. [72]	*Sampling*	"Young athletes participate in various sports"	Fitness tests plus questionnaire	Number sports played
Beese et al. [73]	*Multi-sport*	"Changing sports throughout the year"	LESS	Number sports played
Tripp et al. [74]	*Multi-sport*	–	3D motion analyses	Number sports played
Fahimi et al. [75]	*Diversification*	–	AAHPERD soccer, volleyball and basketball tests	–
Mateus et al. [76]	*Diversification*	–	Batak-Pro Reaction time tests	Number sports played
Santos et al. [24]	*Late specialization*	"Practiced more types of sports during their sporting career"	Questionnaire & coached practice sessions	Number sports played
Miller et al. [77]	*Multisport*	–	Bone mineral density	Number sports played
DiStefano et al. [3]	*Sampling*	–	LESS	Number sports played
DiCesare et al. [2]	*Multisport*	–	Vertical jump- knee angles	Number sports played
Herman et al. [78]	*Multisport*	–	LESS	Number sports played
Salin et al. [79]	*Multisport*	"Engaging in multiple sport activities"	Questionnaire & fitness test	Number sports played
McFadden et al. [6]	*Late specialization*	"Wait until after the age of 12 to focus on a single sport"	questionnaire	Number sports played
Buhrow et al. [80]	*Diversification*	"Participation in practicing, training, or playing in a variety of sports"	Questionnaire	Number sports played
Kearney et al.s [81]	*Multisport*	–	Questionnaire	Number sports played

LESS, landing error scoring system; AAHPERD. American Alliance for Health, Physical Education, Recreation and Dance

#### Quantitative Studies

The 51 remaining quantitative investigations were examined, with particular attention to how terms were defined, operationalised, and measured. Altogether these studies represented 62% of the articles within this review and ranged in publication from 2008 to 2021. Sample type was mixed with 30 studies (59%) focusing on adult athletes (> 18yrs), 20 (39%) involving youth athletes (< 18yrs), and one (2%) that included coaches and parents [81]. Ages of the samples ranged from six years of age [72] to 30 + years [40, 54, 63] and sample sizes ranged from eight participants [67] to over 1000 [44, 51, 57].

Among the 51 quantitative studies, two main themes of research emerged. The first included 37 papers (73%) that investigated the development pathways of athletes within specific sports [67], different levels of sport [68], or different ages [53], with the aim of using the background of elites to understand the pathway to expertise. The second theme included 11 studies (22%) and investigated physiological differences in athletes and used methods like bone mineral density [77], vertical jump knee angles [2], landing error scores [73], fitness tests [72], and reaction time tests [76] to investigate physiological differences between athletes from different engagement pathways. The remaining three studies (6%) investigated mental health [6], mental toughness [80], as well as parental knowledge of the different pathways [81]. General findings, once again, showed benefits to being a multisport athlete from better neuromuscular control [3, 78] and increased bone mineral densities [77], to better reaction times and motor skills [72, 75].

When looking at the terminology within the 58 empirical studies, 50% (*n =* 29) used *diversification* and 29% (*n =* 17) used *sampling*, 14% (*n =* 8) used the term *multisport*, with the remaining 6% (*n =* 4) using the term *late specialization*. Of the studies that focused on physiological differences between different types of athletes, over half used the term *multisport* rather than *diversification* or *sampling*, suggesting the terminology used may be somewhat discipline specific.

### Terminology and Methodology Within Multisport Pathways to Expertise

Of the 37 studies that looked at the developmental background of athletes to determine optimal pathways to expertise, 84% (*n =* 31) used retrospective questionnaires to determine the different pathways athletes may have taken. The remaining six studies in this group (14%) used interviews or website data.

The 37 studies ranged in publication year from 2008 to 2021 with 23 (62%) published in the last five years. Regarding terminology, 62% (*n =* 23) used *diversification*, and 30% (*n =* 11) used *sampling* (the remaining 8%, (*n =* 3), used the terms *multisport* or *late specialization*). There was also widespread (65%, *n =* 24)) interpretation of the multisport pathway as reflecting *more than one sport played across an athlete’s lifetime* versus *within the same year* (32%, *n =* 12) (with no exploration of overlapping seasons within those time frames). Cowan et al. [61] was the only study to consider multiple sports seasonally within the same year, but this was mostly because the sample was alpine skiing and so the seasonal availability of snow created this interpretation. When looking at the definitions of *diversification* and *sampling*, all mentioned involvement in many sports at a young age, but only 38%, (*n =* 14), mentioned elements of “play” or “enjoyment” within their definition.

Regarding methodology, three general approaches emerged within the 31 studies that investigated development pathways using questionnaires; 16 (52%) asked about the number of sports an athlete played, eight (26%) asked about the number of sports an athlete played as well as play versus practice in their main sport, and the remaining seven (23%) asked about play and practice in both the main and other sports. Of the 16 studies that simply asked how many sports an athlete played, the majority (63%, *n =* 10) used the term *diversification*, and 31% (*n =* 5) used *sampling*. Of the remaining 15 studies that focused on ‘play’ in some capacity, the majority of these also used the term *diversification* (53%, *n =* 8), with 33% (*n =* 5) using *sampling* (of the remaining two studies, one used the term *various sports*, and another used both *diversification* and *sampling* interchangeably). Within the eight studies focusing on main sport play and practice and number of other sports, four [16, 49, 61, 66] used the same questionnaire, the “Participant History Questionnaire” (PHQ) developed by Ford et al. [48]. Finally, when considering the 15 studies that investigated play in some capacity, 80% (*n =* 12) were published within the last five years suggesting a change in how the multisport/single sport debate is being explored. However, unlike the conclusion reached earlier that either a multisport or single-sport pathway can lead to high-performance athletes, when looking deeper into the multisport pathway alone, conclusions were mixed. Some studies showed more practice than play resulted in more elite athletes [27, 59], while others showed more play than practice resulted in elite athletes [60]. These mixed results emphasize the importance of clarity in terminology. The mechanisms by which this pathway leads to more high-performance athletes has not been determined, but if different terms are being used, identifying these mechanisms becomes more difficult.

## Discussion

The goal of this review was to investigate the evidence base for multisport pathways in early athlete development, and critically review elements of this pathway relating to terminology and methodology. The majority of the published articles in the area were empirical studies and almost half (45%) of these focused on athletes’ development pathways. Within the samples of these studies was a mixture of high-performance athletes [20], non-elite athletes [16], coaches [23], technical experts [38], senior athletes [63], youth athletes [8], female athletes [66], and male athletes [50] from countries around the world and a range of different sports. Collectively, this descriptive information highlights the diversity and breadth of the research base. There has also been an impressive range of methodologies used, from physiological based examinations [77] to the more commonly used questionnaires [66].

Despite the strong interest in this area, there were clear shortcomings of the existing evidence. One example is the lack of consideration of intensity of practice in measures used in prior work, as well as the efficiency of practice. In most arguments about the risks of specializing in one sport at a young age, the risk is presumably driven by poor load management, but only seven studies in this review questioned training hours or intensities of practice in sports outside of the main sport. In addition, the element of practice efficiency is missing, whereby metrics associated with increased performance could be considered against training hours and intensities to allow for more insight into training loads. As mentioned in the Mosher et al. [18] review of early specialization, there is a lack of consensus regarding how much training is too much. Relatedly, the lack of attention to ‘intensity of engagement’ in studies within this review makes it difficult to determine why a multisport pathway would be superior to a single-sport one.

Another factor limiting the strength of the evidence in this area is the inconsistency in how ‘multisport involvement’ has been defined in prior research. Although much work in this area uses terms like *sampling* and *diversification* as if they were synonyms [30, 69], future work should determine the most appropriate terms for capturing the essence of what is being examined theoretically and conceptually, and then determine the most appropriate way to measure it. Consider the term *diversification* and the lack of clarity around what this term means in sport settings. In economics, diversification as an asset management strategy is a way to reduce risk by putting capital into different streams. In agriculture, crop diversification allows year-round growing without depleting the soil of the same nutritional resource. Both imply a commitment to another element instead of a primary focus in one area, which is similar to how diversification in youth sport implies a division of commitment to more than one sport.

Alternatively, *sampling* suggests a lower level of commitment as a key element of engagement. It implies ‘trying’ rather than ‘committing’ to different sports. In a simple example, an athlete could sample many sports at an initial level with minimal engagement and/or diversify their involvement across several sports with a larger commitment of time and energy. Although prior research has considered these as synonymous, it is not clear that these terms refer to the same thing. Moreover, this distinction may be important because the difference in these terms relates to the quality, intensity, and breadth of engagement in other sports. The confusion between the terms has also given rise to new terminology like *specialised sampling* [56, 65] that appears in more recent articles to describe youth-led play but in only one sport. Stegmann et al. [65] describe *specialized sampling* as an optimal pathway for elite Swiss ice hockey players with moderate amounts of coach-led practice, along with high amounts of informal play, but specifically in one sport. This extends early work on the ‘early engagement approach’ [48], which is defined as “minimal diversity in other sports and high levels of play and practice in the primary domain” (p. 73). Deliberate play as a determinant of future performance has been questioned more recently by Güllich et al. [82] and Barth et al. [83], who found youth-led play to be negligible in predicting later performance, versus Sieghartsleitner et al. [56], who found youth-led play was useful in predicting future performance.

While it is not a requirement to use only a single term when referring to a phenomenon being examined, there should be a clear understanding of what these terms mean, especially since they have the potential to be describing different sport development pathways. The outcomes associated with *diversification* may be different from those of *sampling*, but we will not be able to determine this if terms are used interchangeably and without clear definitions. Results from this review do show common elements between definitions like the concept of more than one sport being played, and that the level of intensity is much lower than specialization and deliberate practice. However, the nuances between the different terms being used to describe the multisport development pathways may be important.

For instance, if the distinction between the two pathways of *sampling* or *specializing* is down to enjoyment and play-like activities, most of the retrospective interviews and questionnaire data collected to date are insufficient to make this distinction. Of the studies examining multisport development pathways, most simply measured the number of sports played in an athlete’s lifetime with the majority focusing on the number of sports athletes played during youth. This suggests critical elements (e.g., inherent enjoyment which is tied to the DMSP’s notion of deliberate play and sampling) have been missed. While ‘number of sports played’ allows researchers to make distinctions between whether an athlete was within a multisport pathway or not, it does not allow for more nuanced distinctions about levels of intensity or commitment to different sports. The difference between *sampling* (i.e., decreased commitment, low intensity, high levels of play in multiple sports) and *diversifying* (i.e., more sustained commitment to multiple sports) may be important for understanding the mechanisms driving the potential for positive effects. Key questions for this area are whether the issue of increased ‘load’, often attributed to specialization, is adequately resolved by increasing the number of sports played as a youth (e.g., there is potential for load to increase or stay the same if the number of sports is simply increased), as is commonly implied by the simplified multisport approach. Alternatively, the benefits of multisport engagement may come from the broader range of learning environments or the type of engagement (e.g., heavily structured versus less structured), in which case, the distinction between *sampling* (e.g., short-term engagement with more flexible structure) and *diversification* (e.g., longer-term, more structured engagement) may be important. Finally, when considering the multisport development pathway with regards to expertise, there is potentially greater opportunity for talent to emerge and develop if an individual is exposed to different sports and skill sets [82].

### A New Framework to Illustrate Terminology Differences

In this section, we use the limitations identified in this review to build a framework to guide future work. In particular, we propose stronger definitions for different forms of engagement on the assumption that these distinctions will be important for clarifying the mechanisms driving any effects. Playing pick-up basketball, street hockey, and 5-a-side soccer in the neighbourhood park with friends, for example, could yield different outcomes than scheduled coach-led practices for soccer, gymnastics, and basketball. The latter may still foster enjoyment and include play-like activities, but there is a clear increase in commitment through the inclusion of scheduled practices. Therefore, we propose a clear distinction be made to separate the terms *sampling* and *diversification* to represent two different trajectories that can be taken under the multisport pathway umbrella. *Diversification* would reflect increased levels of structured practice in multiple sports in an environment requiring greater intensity of engagement, while *sampling* focuses on increased levels of unstructured play in multiple sports in a more playful environment. In this way, a youth athlete who plays informal games of multiple sports with friends in parks epitomises the *sampling* pathway, as play is the focus and different sports are involved. A youth athlete who attends baseball, soccer, basketball, and rugby practices epitomises a *diversification* pathway as there is an increased commitment to these sports, practice is the focus, but participation is still in multiple sports. Regarding the target concept, only five of the 58 empirical studies [39, 40, 43, 44, 63] used the term *sampling* and included play in their definition. While none of the 29 empirical studies that used the term *diversification* implied a more committed approach to practicing multiple sports with eight of those 29 including play in their definition of a diversified athlete. Of the five studies that utilized the target concept of *sampling*, three used questionnaires as their methodology, but only collected data on the number of sports being played, not the intensity of participation. While the seven studies whose methodology asked about intensity in both main and other sports (and therefore conceptualized our key concept) used mostly *diversification* and included play within the definition.

As noted by others [18, 83], the multisport versus specialization dichotomy is too simple to capture the nuances and subtleties of youth engagement in sport. Separating *sampling* and *diversification*, and providing clear definitions, rather than using them synonymously with vague or inconsistent indicators, will add more nuance to our understanding of future development pathways. This over-simplification is already being noted in more recent studies like Stegmann et al. [65] who emphasise the *specialized sampling* pathway in their work. This highlights the different single-sport pathways an athlete can take, with varying levels of engagement and intensity of practice, not dissimilar to the different multisport pathways discussed in this review.

**Fig. 2:**
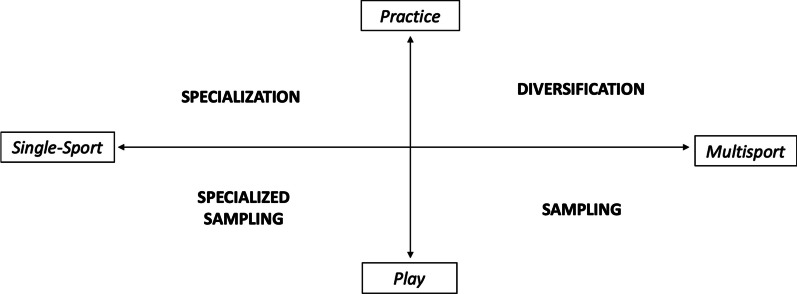
Model showing a vertical axis of engagement intensity, a horizontal axis of number of sports, and how different sport development pathways fit along these axes

Güllich et al. [82] proposed a three-dimensional model to illustrate the differences between *diversification* and *specialization*. However, in their model, *diversification* (defined as multiple sports through youth-led play) was positioned as the polar-opposite to *specialization* rather than *sampling*, even though *sampling* is more commonly tied to play [84]. In Fig. 2, we suggest a model that positions the multisport pathway at the opposite end to the single-sport pathway, along a single versus multisport continuum. Additionally, we suggest another axis, one of commitment, that bisects to make a distinction between the more committed approach of *diversification* (i.e., multiple sports through practice) and the less rigid *sampling* (i.e., multiple sports through play). At the opposite end of the ‘number of sports’ continuum, this axis distinguishes between *specialization* (i.e., one sport through practice) and *specialized sampling* (i.e., one sport predominantly through engagement in play). These divisions provide a more comprehensive framework for determining the range of engagement profiles possible in the multisport pathway. It should also be noted that these axes are both continuums and that an athlete may fit anywhere along them, but these terms are used to describe the general intensity within each quadrant. It is also important to note that the authors are unaware of a term used to describe a single-sport pathway by deliberate play alone that currently exists in the literature. By definition, *specialized sampling* is an oxymoron as *sampling* refers to play in multiple sports, while *specializing* refers to participation in one sport. That said, the operationalization of *specialized sampling* in the literature at present is one sport through mostly play and so is fitting with the model we are proposing. However, a stronger definition for low intensity play within the single-sport pathway is missing.

## Conclusion

The considerable interest in multisport engagement in youth has generated an evidence base with notable breadth. However, despite the range of work in this area, there are critical measurement and conceptual inconsistencies that limit the conclusions that can be made from this evidence, preventing the development of clear guidelines to reduce or eliminate negative effects. By understanding the critical differences between multiple sports through play-focused versus through practice-oriented environments, this can allow greater insight into multi-dimensional development pathways. Furthermore, more investigations into training intensities (e.g., hours per week at coached practiced and games, or months of the year playing sport), frequency of participation (e.g., When are multisport athletes playing different sports? Are they playing multiple sports in a year, in different seasons, or one at a time over their lifetime?) and overall training load (e.g., play and practice) are needed. In addition to greater detail being needed when investigating development pathways, there is a clear need for clarification of terminology. We propose the two most common terms within the multisport development pathway could be differentiated by intensity of play and practice. *Sampling* involves increased play in a less committed and structured environment, while *diversification* reflects increased practice in a more committed and structured environment. This distinction will allow greater insight into potential mechanisms, and greater attention to measurement precision, theoretical consistency and sample heterogeneity will ensure research gaps shrink rather than widen.

## Data Availability

The data that support the findings of this study are available through the Open Source Framework using ID R6W3E.
